# Tumor protein D52 (TPD52) affects cancer cell metabolism by negatively regulating AMPK

**DOI:** 10.1002/cam4.4911

**Published:** 2022-06-06

**Authors:** Yali Chen, Changmin Peng, Wei Tan, Jia Yu, Jacqueline Zayas, Yihan Peng, Zhenkun Lou, Huadong Pei, Liewei Wang

**Affiliations:** ^1^ Department of Oncology Georgetown Lombardi Comprehensive Cancer Center, Georgetown University Medical Center Washington District of Columbia USA; ^2^ Department of Biochemistry and Molecular Medicine The George Washington University School of Medicine and Health Science Washington District of Columbia USA; ^3^ Department of Molecular Pharmacology and Experimental Therapeutics, Mayo Clinic Rochester Minnesota USA; ^4^ Division of Oncology Research, Department of Oncology, Mayo Clinic Rochester Minnesota USA

**Keywords:** AMP‐activated protein kinase (AMPK), cell metabolism, tumor protein D52 (TPD52)

## Abstract

**Background:**

The AMP‐activated protein kinase (AMPK) is a central regulator of energy homeostasis, with deregulation leading to cancer and other diseases. However, how this pathway is dysregulated in cancer has not been well clarified.

**Methods:**

Using a tandem affinity purification/mass‐spec technique and biochemical analyses, we identified tumor protein D52 (TPD52) as an AMPKα‐interacting molecule. To explore the biological effects of TPD52 in cancers, we conducted biochemical and metabolic assays in vitro and in vivo with cancer cells and TPD52 transgenic mice. Finally, we assessed the clinical significance of TPD52 expression in breast cancer patients using bioinformatics techniques.

**Results:**

TPD52, initially identified to be overexpressed in many human cancers, was found to form a stable complex with AMPK in cancer cells. TPD52 directly interacts with AMPKα and inhibits AMPKα kinase activity in vitro and in vivo. In TPD52 transgenic mice, overexpression of TPD52 leads to AMPK inhibition and multiple metabolic defects. Clinically, high TPD52 expression predicts poor survival of breast cancer patients.

**Conclusion:**

The ﬁndings revealed that TPD52 is a novel regulator of energy stress‐induced AMPK activation and cell metabolism. These results shed new light on AMPK regulation and understanding of the etiology of cancers with TPD52 overexpression.

## Graphical abstract

- TPD52 forms a stable complex with AMPKα and negatively regulates AMPKα kinase activity.
- TPD52 modulates cell metabolism through regulating AMPK.

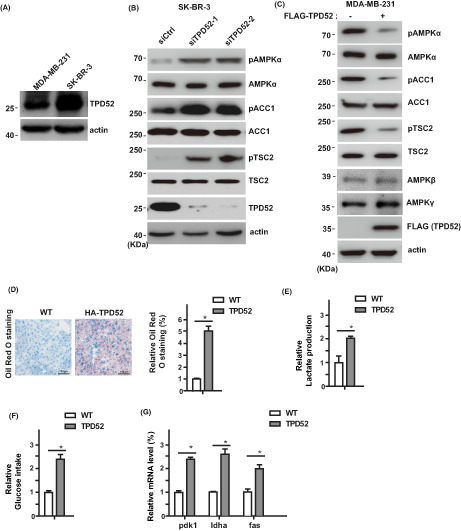

## 1 INTRODUCTION

AMPK (5’‐AMP‐activated protein kinase) is one of the most important molecular energy sensors in eukaryotic cells,^1^, ^2^ which is an evolutionarily conserved heterotrimer formed by a catalytic α subunit and two regulatory subunits (β and γ).^3^, ^4^ The γ subunit contains four cystathionine beta‐synthase (CBS) domains as binding sites for AMP/ADP/ATP.^5^ When cells have an insufficient energy supply, the γ subunit binds to AMP and leads to a major conformational change in the AMPK heterotrimer complex, enabling the exposure of the catalytic pocket of α subunit and activation of AMPK kinase.^6^, ^7^ AMPK activation requires the phosphorylation of Thr172 in α subunit by the upstream kinase liver kinase B1 (LKB1)^8^, ^9^ or CaMKKβ.^10^, ^11^

AMPK is able to control a wide range of metabolic processes that connect cellular metabolism with energy availability.^12^ AMPK controls glucose uptake and fatty acid oxidation in muscle, fatty acid synthesis and gluconeogenesis in the liver, and the regulation of food intake in the hypothalamus.^13^, ^14^, ^15^ AMPK transmits energy crisis signals to downstream targets via phosphorylation events.^16^, ^17^ For example, AMPK phosphorylates acetyl‐CoA carboxylase 1 (ACC1) and sterol regulatory element‐binding protein 1c (SREBP1c) to suppress lipid and cholesterol synthesis^18^, ^19^; AMPK phosphorylates ULK1 to modulate autophagy, which helps cells to adapt to cellular energetic status^20^, ^21^; AMPK phosphorylates TSC2 to inhibit Rheb GTP loading and renders mTORC1 inactivation.^22^

The LKB1‐AMPK pathway is a central regulator of energy metabolism, and dysregulation of this pathway has been implicated in cancers.^23^, ^24^, ^25^ Indirect AMPK activators such as metformin have shown a beneficial effect in breast cancer prevention and treatment.^26^, ^27^ However, other than LKB1 mutations, this pathway might be dysregulated in cancer remains unclear. Therefore, it will be important to identify additional novel regulators in the LKB1‐AMPK pathway that may be involved in cell metabolism abnormalities.

In the present study, we identified TPD52 as a novel regulator of the LKB1‐AMPK pathway. TPD52 is known to be involved in the regulation of vesicle trafficking and exocytotic secretion.^28^, ^29^ In addition, the 8q21 locus containing the *Tpd52* gene is frequently amplified in tumors.^30^, ^31^, ^32^ However, the exact mechanisms of TPD52 in cancer metabolism remain unclear. Our results demonstrated that TPD52 affects cancer cell metabolism by negatively regulating AMPK.

## 2 MATERIALS AND METHODS

### 2.1 Cell culture

HEK293T cells were maintained in a DMEM medium supplied with 10% FBS at 37°C with 5% CO_2_. Human breast carcinoma cell lines (SK‐BR‐3, MCF‐7, MDA‐MB‐231, and T‐47D) were cultured in an RPMI‐1640 medium supplemented with 10% FBS, 6 mM L‐glutamine, and 10 μg/ml insulin (for MCF‐7) at 37°C with 5% CO_2_.

### 2.2 Tpd52 transgenic mice

*Tpd52* transgenic founder mice were generated by Cyagen Biosciences Inc. The PiggyBac transposon gene expression vector was created by cloning the ORF of mouse *Tpd52* gene (*mTpd52*) with an HA‐tag into the Cyagen basic PiggyBac (PB) vector. The pPB‐CAG‐*mTpd52* ORF‐HA was linearized and purified for pronucleus injection. The fertilized one‐cell eggs from the superovulated female C57BL/6 mice were injected and implanted into the oviducts of pseudopregnant female C57BL/6 mice. The positive founders were screened by genotyping using primers (forward 5’‐CTGGTTATTGTGCTGTCTCATCAT‐3′ and reverse 5’‐TCAGCAGACCAACGTTCTGTG‐3′) with a product of 214‐bp fragment. The identified positive founders were then bred with wild‐type C57BL/6 mice for the generation of the *mTpd52* transgenic mice.

### 2.3 Constructs, antibody, and siRNA

S/FLAG/SBP‐tagged TPD52 was generated in the pIRES2‐EGFP vector. HA‐tagged TPD52 was generated in the pCMV‐HA vector. TPD52 cDNA was cloned in pGEX‐4 T‐2 to generate GST fusion protein expression plasmids. His‐tagged AMPKα, β, and γ expression constructs were generated from pET‐28a. TPD52 deletion mutants were generated using site‐directed mutagenesis (Stratagene).

The following antibodies were used: phospho‐AMPKα (2535S), AMPKα (5832S), AMPKβ (4150S), AMPKγ (4187S), phospho‐ACC1 (3661S), ACC1 (3662S) from CST, TPD52 (GTX115042) from GeneTex, anti‐His tag (sc8036) from Santa Cruz, anti‐FLAG (F7425, F3165) from Sigma‐Aldrich, and anti‐HA (901501) from Biolegend.

siRNAs against TPD52 (Thermo Fisher Scientific) were as follows: 5’‐GCGGAAACUUGGAAUCAAU‐3′ (siTPD52‐1) and 5’‐GGAGAAGUCUUGAAUUCGG‐3′ (siTPD52‐2). Nontargeting siRNA (All‐star negative control siRNA) was purchased from QIAGEN.

### 2.4 Immunoprecipitation assay

Cells transfected with indicated constructs were lysed in NETN buffer (10 mM Tris–HCl, pH 8.0, 100 mM NaCl, 1 mM EDTA, and 0.5% NP‐40) plus protease inhibitor (Roche) on ice for 30 min. Then cell lysates were incubated with indicated beads (FLAG M2 or HA beads from Sigma‐Aldrich) for 8 h at 4°C. For endogenous IP, cell lysates were incubated with indicated antibodies and Protein A/G beads (Thermo Fisher Scientific). The immunocomplexes were washed with NETN buffer three times and applied to SDS‐PAGE. Immunoblotting was performed by the following standard procedures.

### 2.5 Protein purification

Tandem affinity purification was performed to purify the TPD52‐associated complex. Briefly, HEK293T cells transfected with S/FLAG/SBP‐tagged TPD52 were lysed with NETN buffer plus protease inhibitor (Roche), then incubated with streptavidin sepharose beads (Amersham Biosciences) for 1 h at 4 °C. The bound proteins were washed and then eluted with 2 mM biotin (Sigma) for 30 min twice at 4°C. The eluates were incubated with S‐protein agarose (Novagen) for 1 h at 4°C and then washed. The bound proteins were resolved by SDS–PAGE and visualized by Coomassie blue staining. The identities of eluted proteins were revealed by mass spectrometry.

GST fusion and His‐tagged proteins were expressed and purified from *Escherichia coli* BL21 bacteria. The cell lysates supernatant were immobilized on Glutathione Sepharose 4B (GE healthcare) or Ni‐NTA (Thermo Fisher Scientific) at 4°C overnight. According to the manufacturer's instructions, proteins were then eluted and stocked in PBS containing 5% Glycerol at −80°C.

### 2.6 GST pull‐down assay

HEK293T cells were transfected with indicated constructs. After 48 h, cells were lysed in NETN buffer plus protease inhibitors and were incubated with the GST fusion proteins‐loaded Glutathione Sepharose 4B beads at 4°C for 6 h. The beads were washed with NETN buffer five times. The bound proteins were applied to SDS‐PAGE and immunoblotting as above.

### 2.7 mRNA extract and qRT‐PCR

Total mRNA was isolated and reverse transcribed using FastKing RT kit (Tiangen) according to the manufacturer's protocol. Real‐time PCR was performed with the following primers: mouse *actin* (forward 5′‐CGGTTCCGATGCCCTGAGGCTCTT‐3′ and reverse 5′‐ CGTCACACTTCATGATGGAATTGA‐3′), mouse *ldha* (forward 5′‐TGTCTCCAGCAAAGACTACTGT‐3′ and reverse 5′‐ GACTGTACTTGACAATGTTGGGA‐3′), mouse *pdk1* (forward 5′‐ACAAGGAGAGCTTCGGGGTGGATC‐3′ and reverse 5′‐ CCACGTCGCAGTTTGGATTTATGC‐3′), mouse *fas* (forward 5′‐GCTGCGGAAACTTCAGGAAAT‐3′ and reverse 5′‐ AGAGACGTGTCACTCCTGGACTT‐3′), and mouse *scd1* (forward 5′‐CTGACCTGAAAGCCGAGAAG‐3′ and reverse 5′‐ GCGTTGAGCACCAGAGTGTA‐3′).

### 2.8 Oil Red O staining

The intracellular lipid droplet contents of cultured cells were evaluated by Oil Red O staining. Briefly, cells were washed with ice‐cold PBS, fixed with 10% formalin for 60 min, and stained with Oil Red O working solution (1.8 mg/ml of Oil Red O in 6:4 isopropanol:water solution) for 60 min at 25°C. Cells were washed with water to remove any remaining dye. For quantification of Oil Red O staining, the cell‐retained dye was extracted by isopropanol and the content was measured spectrophotometrically at 500 nm.

### 2.9 Animal diet

WT and transgenic male mice at the age of 6 weeks were fed on a nonfat diet (NFD) or high‐fat diet (HFD, D12492, QiFa Biotech.) for 16 weeks. GTT assay was performed in 16‐week‐old mice. Blood glucose level was performed in 20‐week‐old mice. IHC and Oil Red O staining and qRT‐PCR were performed with liver tissue samples from 22‐week‐old mice. These procedures were approved by the Institutional Animal Care and Use Committee (IACUC).

### 2.10 Glucose consumption and lactate production

The indicated constructs were transfected in MDA‐MB‐231 cells. After 48 h, cells were trypsinized and counted, while the supernatants of the cell culture medium were collected. The media were assayed immediately for glucose and lactate levels by using a glucose assay kit and lactate assay kit (Biovision) according to the manufacturer's instruction. The glucose consumption and lactate production were normalized to cell numbers.

### 2.11 Liver histological and immunohistochemical analysis

Livers were fixed in 10% phosphate‐buffered formalin acetate at 4°C overnight and embedded in paraffin wax. Paraffin sections (5 μm) were cut and mounted on glass slides for hematoxylin and eosin (H&E) staining. Cryosections of livers were stained with Oil Red O and counterstained with hematoxylin to visualize the lipid droplets. Immunohistochemistry of liver sections was also performed with indicated antibodies.

### 2.12 Blood glucose detection

Mice were fasted for 16 h. Blood glucose levels were measured in tail vein blood samples using a glucometer. Values are expressed as mean ± SD (*n* = 6).

### 2.13 IPGTT (glucose tolerance test)

After 5 h of fasting, male mice were injected with 1 g/kg of bodyweight glucose intraperitoneally. Blood glucose was measured before the glucose injection and at 15, 30, 60, and 90 min postinjection. Values are expressed as mean ± SD (*n* = 6).

### 2.14 Statistical analysis

All the other statistical analysis was performed with data from three biological triplicates. Statistical analysis was performed by the Student's *t* test for two groups and by ANOVA for multiple groups. *p* < 0.05 was considered significant.

## 3 RESULTS

### 3.1 TPD52 interacts with AMPKα

**FIGURE 1:**
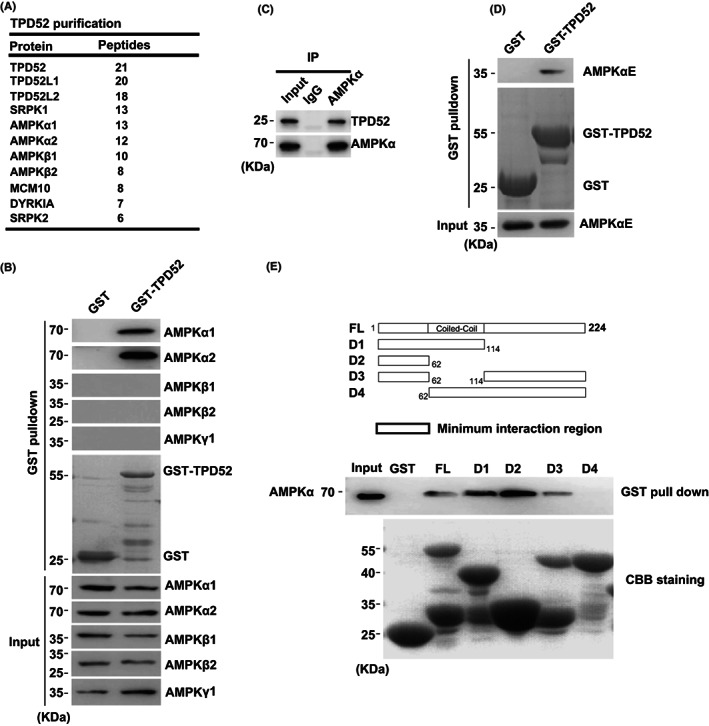
TPD52 interacts with AMPKα. (A) Tandem affinity purification was performed using HEK293T cells stably expressing N‐FLAG‐S‐tagged TPD52. The major hits from mass spectrometry analysis were shown in the table. (B) His‐tagged AMPKα1, α2, β1, β2, and γ1 were overexpressed and purified from *Escherichia coli*, and a GST pull‐down assay using GST‐TPD52 was carried out to determine the in vitro interactions. (C) TPD52 interacts with AMPK endogenously in 293T cells. Irrelevant IgG was used as the immunoprecipitation control. The whole‐cell lysate was used as an input. (D) His‐tagged AMPKα1 kinase domain (AMPKαE) was overexpressed and purified from *E*. *coli*, and a GST pull‐down assay of TPD52 using the purified proteins was carried out to determine the interactions. (E) Mapping the regions of TPD52 required for AMPK binding. Upper panel, schematic representation of TPD52 constructs and the minimum interaction region. Lower panel, GST‐tagged TPD52 full‐length (FL) or deletion mutants were purified from *E*. *coli*, and a GST pull‐down assay was performed.

TPD52 is known to be commonly overexpressed in breast cancer, but the physiological functions are not clear. To gain greater insight into the function of TPD52, tandem affinity purification using FLAG‐S‐TPD52‐overexpressed 293T cells was performed (Figure 1A). We identified AMPK (including α1, α2, β1, and β2 subunits) as a major TPD52‐associated protein. Some known TPD52‐associated proteins were also copurified, including TPD52L1, TPD52L2, SRPK1, and SRPK2. We confirmed this interaction using an in vitro binding assay with purified His‐tagged AMPK subunits and GST‐TPD52. TPD52 is specifically bound to AMPK α1 and α2, but not the other subunits (Figure 1B). In addition, purification of endogenous AMPKα also identified TPD52 as a major AMPK‐associated protein (Figure 1C). We also determined the interactions between TPD52 and AMPKα1 kinase domain (1–300 amino acids, AMPKαE) using a GST‐pull‐down assay (Figure 1D). Furthermore, we characterized the N‐term region (amino acid residues 1–61) of TPD52 was responsible for its interaction with AMPKα (Figure 1E).

### 3.2 TPD52 regulates AMPK activation and cellular metabolism

**FIGURE 2:**
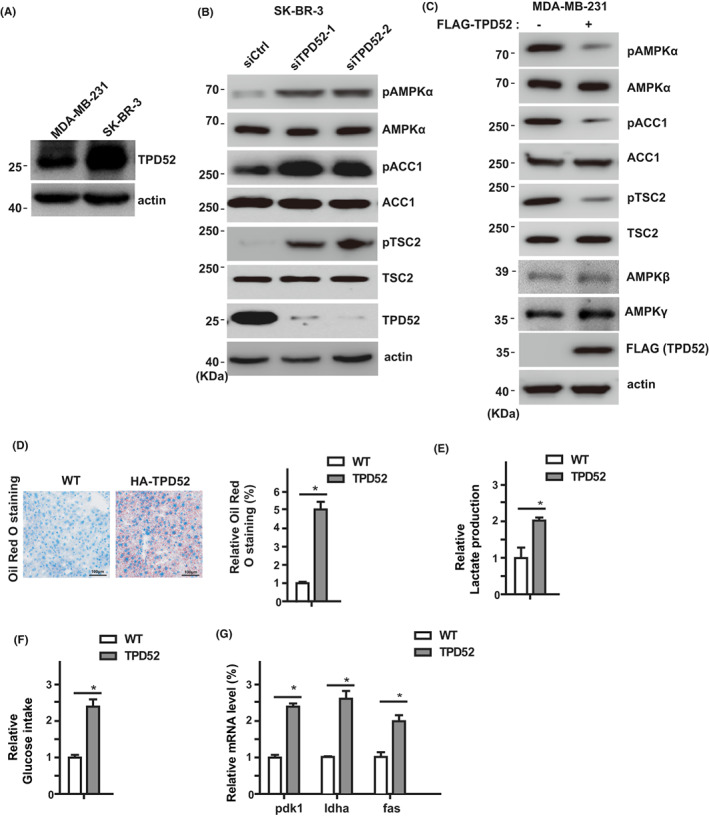
TPD52 regulates AMPK activity and cellular metabolism. (A) SK‐BR‐3 cells were transfected with indicated siRNAs. The phosphorylations of AMPKα, ACC1, and TSC2 in cell lysates were detected by Western Blot. (B) MDA‐MB‐231 cells were transfected with the indicated constructs, and the phosphorylations of AMPKα, ACC1, and TSC2 in cell lysates were detected by Western Blot. (C) MDA‐MB‐231 cells were transfected with the indicated constructs, and the intracellular lipid droplet level was assessed by Oil Red O staining. All error bars represent the SD from the mean value of three independent experiments. *p < 0.05. (D) Overexpression of TPD52 increases lactate production. The lactate production was measured in a medium collected at 48 h after transfection of control or TPD52 expressing constructs in MDA‐MB‐231 cells. The results represent the mean ± SEM of three independent experiments. *p < 0.05. (E) Overexpression of TPD52 increases glucose intake. The glucose intake was measured in a medium collected at 48 h after transfection of control and TPD52 overexpressing constructs in MDA‐MB‐231 cells. The results represent the mean ± SEM of three independent experiments. *p < 0.05. (F) Overexpression of TPD52 increases lipogenesis and glycolytic gene expression. Relative expression levels of *fas*, *ldha*, and *pdk1* mRNA in control or TPD52 overexpressed MDA‐MB‐231 cells were determined by qPCR. Transcript levels were determined relative to actin mRNA levels and normalized to control cells. The results represent the mean ± SEM of three independent experiments. *p < 0.05.

Since TPD52 interacts with AMPKα, we next investigated whether TPD52 could regulate AMPK activation. Knockdown of TPD52 in SK‐BR‐3 cancer cells resulted in increased phosphorylation of AMPKα at Thr172, while the total AMPKα level was not affected (Figure 2A). Conversely, overexpression of TPD52 in MDA‐MB‐231 resulted in decreased AMPK Thr172 phosphorylation (Figure 2B). ACC1 and TSC2 were well‐established substrates of AMPK. We also found that knockdown of TPD52 significantly increased the phosphorylation of ACC1 and TSC2 (Figure 2A), whereas overexpression of TPD52 decreased the phosphorylation of ACC1 and TSC2 (Figure 2B). These results suggest that TPD52 negatively regulates AMPKα activation in vivo.

AMPK plays an important role in cellular metabolism.^33^, ^34^ To investigate whether TPD52 regulates AMPK‐dependent metabolic processes, we examined cellular lipid metabolism and glycolysis. TPD52 overexpression resulted in significant increases in lipid drop formation, lactate production, and glucose intake (Figure 2C–E) and also increased the expression of glycolytic genes (such as *pdk1*, *ldha*, and *fas*) (Figure 2F). Meanwhile, TPD52 deficiency resulted in significant decreases in lipid drop formation, lactate production, and glucose intake (Figure S1A–C). All these metabolic changes further supported that TPD52 regulates the AMPK pathway and cellular metabolism.

### 3.3 TPD52 directly regulates AMPK kinase activity

**FIGURE 3:**
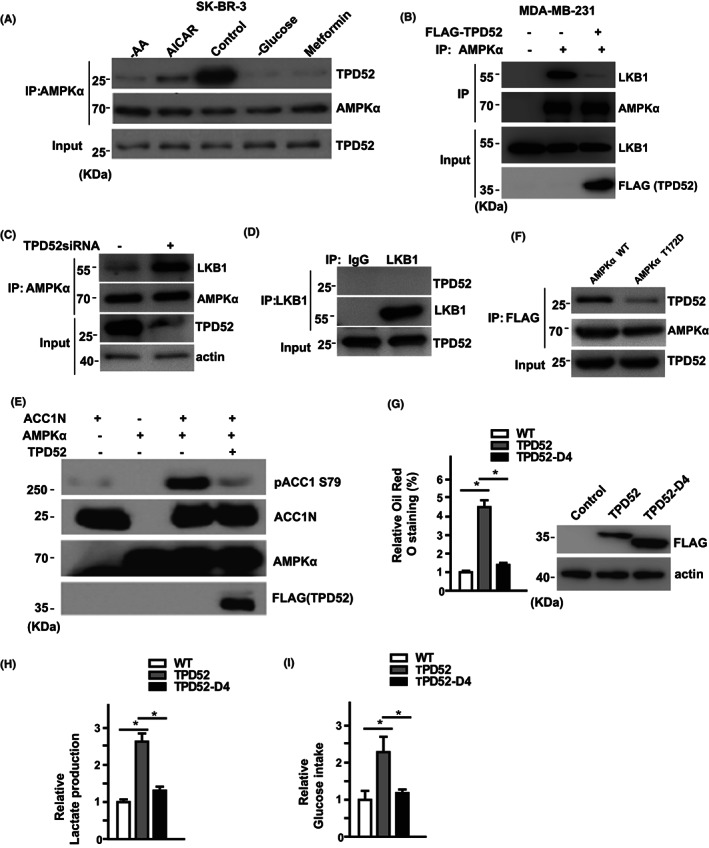
TPD52 directly regulates AMPK kinase activity. (A) SK‐BR‐3 cells were treated as indicated for 24 h, and the interaction between TPD52 and AMPKα was examined by co‐IP. (B) MDA‐MB‐231 cells were stably transfected with TPD52, and LKB1–AMPKα interaction was examined by co‐IP. (C) TPD52 directly inhibits ACC1 Ser79 phosphorylation by AMPKα in vitro. GST‐TPD52, AMPKα, and ACC1N were purified from *E*. *coli* as indicated in the Methods, and an in vitro kinase assay was performed. (D) MDA‐MB‐231 cells were transfected with the indicated constructs, and the intracellular lipid droplets were assessed by Oil Red O staining. All error bars represent the SD from the mean value of three independent biological replicates. (E) The lactate production was measured in a medium collected at 48 h after transfection of control or TPD52 WT or D4 mutant expression constructs in MDA‐MB‐231 cells. The results represent the mean ± SEM of three independent experiments. *, *p* < 0.05. (F) The glucose intake was measured under the same condition as the previous ones. The results represent the mean ± SEM of three independent experiments. *p < 0.05.

As AMPK is activated by many nutrition stresses, we next examined whether these nutrition stresses might affect AMPK–TPD52 interaction. Treatments like pharmacological AMPK activators (AICAR or metformin), amino acid shortage (−AA), or glucose deprivation (−Glucose) all dramatically decreased AMPK–TPD52 interaction (Figure 3A), implying that the TPD52–AMPK axis is an important in cell response to nutrition stresses. Given that TPD52 regulated AMPK activation (Figure 2), we examined the mechanisms by which TPD52 regulated AMPK activity. It is possible that the binding of TPD52 to AMPKα influences the interaction between AMPK with its upstream kinase LKB1. To test it, we performed co‐IP and found that TPD52 overexpression blocked the AMPK–LKB1 interaction (Figure 3B). It is also possible that TPD52 has a direct effect on AMPK kinase activity. To test this, we constructed an in vitro kinase assay with purified ACC1 N terminal (1–200 amino acids, ACC1N) as AMPK substrates. We found that TPD52 directly inhibited the AMPK‐mediated phosphorylation of ACC1N (Figure 3C). These results suggest that TPD52 affects AMPK activity by dual mechanisms as follows: 1, by affecting LKB1–AMPKα interaction and, in turn, AMPK Thr172 phosphorylation and 2, by directly inhibiting AMPK kinase activity toward its substrates.

Since TPD52 directly interacts with AMPKα through its N terminal, we hypothesized that TPD52–AMPK interaction is required for TPD52 functions in cell metabolism. In contrast to WT TPD52, overexpression of interaction‐deficient D4 mutant failed to affect lipid drop formation, lactate production, and glucose intake (Figure 3D–F). These results indicated that TPD52 regulated AMPKα activity and cell metabolism through direct interaction with AMPK.

### 3.4 TPD52 regulates AMPK activity and cell metabolism in vivo

**FIGURE 4:**
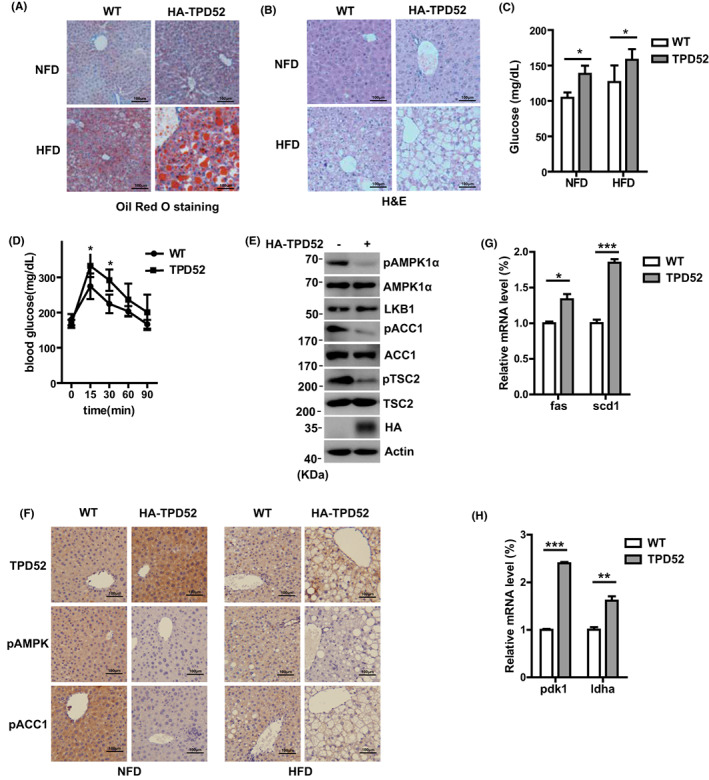
TPD52 regulates AMPK activity and cell metabolism in vivo. (A, B) The Oil Red O staining and H&E staining of the liver tissues from the NFD‐ and HFD‐fed WT and HA‐TPD52 transgenic mice. (C) Blood glucose levels were measured in tail vein blood samples using a glucometer. Values are expressed as mean ± SD (*n* = 6). *p < 0.05. (D) Serial changes in blood glucose levels after intraperitoneal injection of glucose in the indicated mice (*n* = 6). Values are expressed as mean ± SD. *p < 0.05. (E) AMPK phosphorylation, precursor (−p), and nuclear‐processed (−n) SREBP1c and HA‐TPD52 levels in liver tissues from the indicated mice were examined by Western Blot. (F) Expression of TPD52 and phosphorylated ACC1 and AMPK in livers from the NFD‐ and HFD‐fed WT and HA‐TPD52 transgenic mice was determined by IHC staining. (G, H) mRNA levels of lipogenesis gene (*fas* and *scd1*) and glycolytic gene (*pdk1* and *ldha*) in indicated liver cells from the WT and HA‐TPD52 transgenic mice were determined by qRT‐PCR. Relative mRNA levels were corrected to actin mRNA levels and normalized relative to control cells. The results represent the mean ± SEM of three independent experiments. ***p < 0.001, **p < 0.01, *p < 0.05.

To explore the function of TPD52 in vivo, we generated *Tpd52* transgenic mice (Figure S2A). *Tpd52* overexpression in these mice was confirmed through genotyping and Western Blot (Figure S2B, C). We noticed that these mice showed multiple metabolic defects, whether under NFD or HFD conditions. TPD52 overexpression significantly increased liver lipid contents, especially under the HFD condition, as measured by Oil Red O staining and H&E staining (Figure 4A,B). In addition, TPD52 overexpression also resulted in increased blood glucose levels under both NFD and HFD conditions (Figure 4C) and lower glucose tolerance (under NFD conditions, Figure 4D). To further clarify whether these phenotypes were related to AMPK, we measured the activation status of AMPK in the liver tissues. Consistent with cell‐based assay results, TPD52 overexpression resulted in a drastic decrease in phosphorylation of AMPKα (p‐AMPKα) and a more nuclear active form of SREBP1 (SREBP1‐n) in livers (Figure 4E). We also observed a decrease in phosphorylation of ACC1 (Figure 4F) in the livers of the TPD52 transgenic mice, indicating that AMPK is less activated. Expression levels of glycolytic genes, such as *scd1*, *fas*, *pdk1*, and *ldha*, were significantly increased in TPD52 transgenic mice (Figure 4G,H). These phenotypes were consistent with the function of AMPK in glycolysis and lipogenesis.^34^, ^35^

**FIGURE 5:**
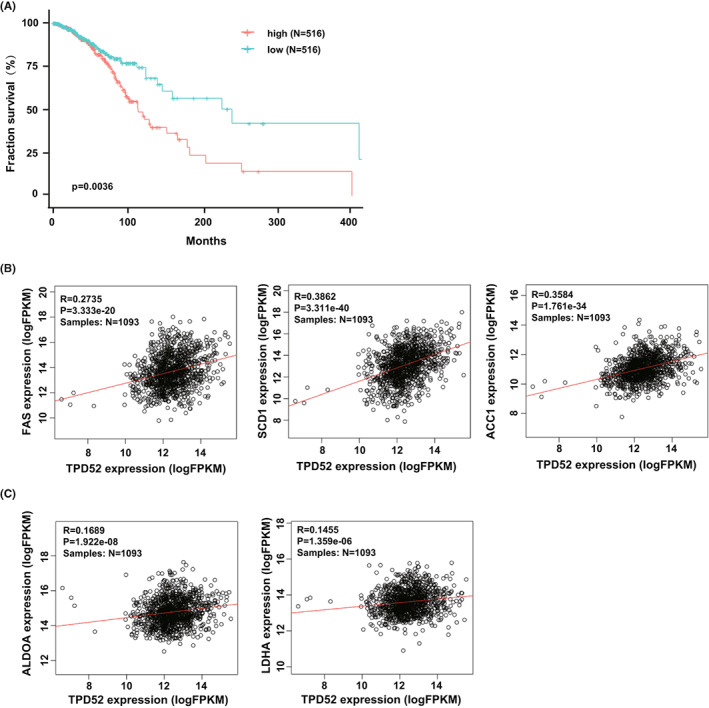
TPD52 expression is associated with clinical outcome and metabolic gene expression in breast cancer patients. (A) Kaplan–Meier (KM) survival analysis of the breast invasive carcinoma patients in TCGA cohort (https://portal.gdc.cancer.gov, TCGA‐BRCA, V13.0, 2018). The patients were divided into high‐ and low‐expression groups based on the medium FPKM cutoff value of the TPD52 level. **p < 0.01. (B) TPD52 and the lipogenesis genes (*fas*, *scd1*, and *acaca/acc1*) are coexpressed in breast invasive carcinomas. The correlation was calculated using Pearson's correlation coefficients (R). (C) TPD52 and the glycolysis genes (*aldoa* and *ldha*) are coexpressed in breast invasive carcinomas. The correlation was calculated using Pearson's correlation coefficients (R).

The protein expression profiles from the Human Protein Atlas have revealed that TPD52 is upregulated in the majority of breast cancer tissues. To determine whether TPD52 expression correlates with patient outcome, we analyzed the overall survival (OS) with a TCGA‐BRCA cohort of 1109 Breast invasive carcinoma samples. Indeed, the log‐rank test demonstrated that the overall survival for patients with low TPD52 expression in tumor tissues was significantly higher than those with high TPD52 expression (*p* = 0.0036, Figure 5A), suggesting that TPD52 expression correlates with poor patient prognosis. We also used the RNA‐seq data set of the above TCGA‐BRCA consortium to analyze the coexpression profiles between TPD52 and the metabolic genes. We observed that the expression profiles of TPD52 and the lipogenesis genes such as FAS, SCD1, ACACA/ACC1 were significantly correlated (R = 0.274, 0.386, 0.358, respectively, Figure 5B), which is consistent with our in vivo data from mice. We also found a positive correlation between the expression profiles of TPD52 and the AMPK downstream glycolysis genes such as Aldoa and Ldha (R = 0.169, 0.146, respectively, Figure 5C), indicating a negative correlation between TPD52 expression level and AMPK activity.

Taken together, our results suggest that TPD52 plays a key role in AMPK activation and controls lipid and glucose metabolism in vivo.

## 4 DISCUSSION

The LKB1–AMPK pathway has been identified as a central regulator of energy metabolism. Many reports have demonstrated molecules regulating the AMPK pathway by different mechanisms. However, there is none reported to regulate AMPK through direct interaction. In this study, we have established TPD52 as a regulator of energy stress‐induced AMPK activation and cell metabolism. TPD52 is directly bound to AMPKα, and TPD52 binding blocks the LKB1–AMPK interaction. It is possible that TPD52 and LKB1 might bind to the same regions of the catalytic domain of AMPKα coordinately to regulate AMPK phosphorylation. Thereby nutrition or energy stress disrupted AMPK–TPD52 interaction and facilitated AMPK–LKB1 interaction. On the other hand, TPD52 also regulates AMPK‐mediated phosphorylating ACC1 in vitro. The conformation change of the kinase domain of AMPKα is very important for AMPK activation.^7^, ^36^ TPD52 binding might directly influence AMPKα confirmation and, in turn, affect its kinase activity. These all need further validation with certain structural biology techniques.

Meanwhile, AMPK can also be activated by CaMKKβ in response to calcium flux.^10^, ^11^ It would be interesting to test whether TPD52 also functions in cell response to calcium flux in future studies.

Dysregulation of the LKB1–AMPK pathway has been implicated in many cancers including breast cancer.^23^, ^24^, ^25^ However, other than LKB1 mutations, how this pathway might be dysregulated in cancer remains unclear. The chromosome 8q21 locus containing the *Tpd52* gene is frequently amplified in multiple tumors. TPD52 has been characterized as a potential biomarker and therapeutic target in tumors, but its working mechanisms are not yet elucidated. Here we found that TPD52 regulates the AMPK pathway and cell metabolism in vitro and in vivo. *Tpd52* transgenic mice showed multiple metabolic defects, which mimics many phenotypes of AMPK knockout mice.^37^ By exploring TCGA‐BRCA cohort data, we also found that TPD52 expression is associated with clinical outcomes and metabolic gene expression in breast cancer patients. As cell metabolism dysregulation is a key characteristic for tumor initiation and progression. Our studies provided new insights into TPD52‐associated cancer etiology.

Our findings will have a significant impact on the dissection of components in the pathway controlling AMPK activity. As AMPK plays an important role in lots of cell‐signaling pathways,^16^, ^17^ it could be an ideal therapeutic target for the treatment of obesity, insulin resistance, type 2 diabetes, and cancer.^38^, ^39^ Our findings may also have important implications for multiple disease etiology and therapies.

## AUTHORS' CONTRIBUTION

Conceptualization: H.P.; Methodology: Y.C., C.P., W.T., J.Y., J.Z., and Y.P.; Investigation: Y.C., W.T., J.Y., and J.Z.; Writing–Original Draft: H.P.; Writing–review and editing: Z.L., L.W., and H.P.; Funding Acquisition: H.P., Y.C., and L.W.; Supervision: Z.L., L.W., and H.P. All authors discussed the results and comments on the manuscript.

## FUNDING INFORMATION

L.W. and Z.L. were funded by the NCI (grant no. CA196648).

## DISCLOSURE STATEMENT

All authors have no competing interests to declare, financial or otherwise.

## ETHICS APPROVAL STATEMENT

All animals were handled in strict accordance with the “Guide for the Care and Use of Laboratory Animals” and the “Principles for the Utilization and Care of Vertebrate Animals,” and all animal work was approved by the Institutional Animal Care and Use Committee of the George Washington University (USA).

## Supporting information


Figure S1‐S2

## Data Availability

The authors declare that all data supporting the findings of this study are available within the paper and its Supplementary Information files.
